# Primary pleural epithelioid hemangioendothelioma: case report and review of the literature

**DOI:** 10.1097/CAD.0000000000001122

**Published:** 2021-07-04

**Authors:** Daniele Lavacchi, Luca Voltolini, Camilla Eva Comin, Francesca Mazzoni, Giacomo Giulio Baldi, Vittorio Briganti, Silvia Luvarà, Stefano Bongiolatti, Lorenzo Antonuzzo

**Affiliations:** a Clinical Oncology Unit; b Thoracic Surgery Unit, Careggi University Hospital; c Department of Experimental and Clinical Medicine, University of Florence, Florence; d Department of Medical Oncology, Hospital of Prato, Azienda USL Toscana Centro, Prato; e Division of Nuclear Medicine, Careggi University Hospital; f Department of Emergency Radiology, Careggi University Hospital, Florence, Italy

**Keywords:** antiangiogenic drugs, epithelioid hemangioendothelioma, extrapleural pneumonectomy, immunomodulatory drugs, pleural EHE

## Abstract

Epithelioid hemangioendothelioma (EHE) is an extremely rare vascular sarcoma with an unpredictable clinical behavior. Pleural EHEs have been associated with poor response to treatment and reduced survival. To date, no standard treatment for EHE is available. Here we report the case of a 53-year-old man who underwent radical surgery for a symptomatic primary pleural EHE. Clinical presentation was characterized by chronic pain in the left hemithorax with transitory flare, anemia, weight loss and progressive worsening of clinical conditions. After surgery, he resumed active life and normal daily activities and, at 8 months, 18F-FDG PET and computed tomography scan showed no radiological evidence of recurrent disease. Clinical signs of this rare disease, histological features, imaging findings and functional imaging are discussed. We also report a summary of other cases with resected pleural EHE and we briefly review the role of chemotherapeutic, immunomodulatory and antiangiogenic drugs for advanced disease.

## Introduction

Epithelioid hemangioendothelioma (EHE) is an extremely rare vascular sarcoma, affecting approximately less than one in a million people worldwide. The clinical courses of patients with EHE are characterized by initial misdiagnoses and unpredictable behavior [1,2].

Although different primary tumor locations have been described so far, more frequently EHE arises in liver, soft tissue or lung, with a typical multifocal and/or multicentric onset. Pleura is an uncommon site for primary tumor and [3,4] pleural involvement may be mostly a result of the secondary spread of intrathoracic or extrathoracic EHEs. Pleural primary location and the presence of pleural effusion are commonly considered worst prognostic factors and are associated with worse survival [1,5–9]. Pleural involvement is characterized by fibrinofibrose pleural lesions with millimeter thickness, sometimes forming a fibrothorax with serosal and interstitial spread [1,4]. Other presenting systemic symptoms are long-lasting stabbing pain, poorly responsive to opioids, weight loss, anemia, fever and worsening of clinical conditions [10] (Table 1). Lung involvement in EHE is characterized by small, bilateral, multiple, perivascular nodules without mediastinal nodal metastases in most cases. An indolent course is typically associated with the involvement of parenchymatous organs (e.g. liver-only disease) [1,4].

**Table 1:** Summary of cases with resected pleural epithelioid hemangioendothelioma

Authors	Age and sex	Symptoms at presentation	Signs at presentation	Diagnosis	Surgery	Other treatments	Median DFS from surgery (months)	Median OS from surgery (months)
Lee *et al*. [3]	31 years, F	Upper back and radiating, bilateral shoulder pain.	Right nodular pleural thickening with several foci, and bone metastases.	Thoracoscopy: multiple scattered subpleural whitish-tan plaques; intraparenchymal growth.	Wedge resection of the right lower lobe of the lung	Palliative radiotherapy on the spine and chemotherapy with adriamycin as first-line treatment followed by mesna-doxorubicin-ifosfamide-dacarbazine as second-line treatment	NR	≈10
Crotty *et al*. [4]	4 males with median age of 62 years	Chest pain, dyspnea, productive cough, fever and weight loss	Moderate-sized pleural effusions with smooth and nodular pleural thickening.	Three patients underwent open thoracotomy with pleural decortication.	Three out of four patients underwent pleural stripping and decortication procedures, for diagnostic purposes and palliation.	Not specified	NR	≈10
			Two patients presented multiple pulmonary nodules (ranged from 3 to 12 mm). Diffuse mediastinal lymphadenopathy and thickened interlobular septa were observed in one patient.	One underwent video-assisted thoracoscopic pleural biopsy.				
				Lung was noted to be encased by dense white-gray tissue in one. In another one, a thick white plaque invaded the diaphragm.				
			No chest wall invasion was reported.	Thoracocentesis: negative findings in one patient and positive findings for undifferentiated malignancy in another one.				
Kim *et al*. [11]	46 years, F	Right-sided chest discomfort and cough.	Right-sided pleural effusion	Diagnostic thoracoscopy: thick peel involving the pleural surfaces.	Initial decortication and visceral pleurectomy. Subsequent complete pleurectomy with cytoreduction to minimal disease	Postsurgical chemotherapy with carboplatin and etoposide for residual disease	≈22	≈22
				Thoracocentesis: serosanguineous exudative effusion negative for malignancy.				
Saqi *et al*. [12]	37 years, M	Progressive dyspnea and right-sided pleuritic chest pain	Right-sided pleural effusion	Thoracentesis and pleural decortication	Pleural decortication	Postoperative carboplatin, etoposide, and bevacizumab for one course	NR	≈3
Al-Shraim *et al*. [13]	51 years, M	Dry cough, shortness of breath	Left-sided pleural effusion. Pleural thickening forming a nodular mass	Pleuroscopy: results interpreted as malignant mesothelioma, undifferentiated, small cell type. Correct diagnosis came from histological review	Left lung decortication and resection of the pleural tumor	Interferon	NR	>24
Yu *et al*. [14]	39 years, F	Progressive dyspnea	Mass lesion in the left thorax invading the pericardium and compressing the myocardium	Thoracocentesis and pericardiocentesis: bloody exudative effusion.	Radical resection	Postsurgical chemotherapy with carboplatin and etoposide	>14	>14
				Fine needle aspiration: atypical inflammatory cells.				
				Sternotomy: parenchymatous mass contiguous with a calcified mass on the left pleura.				
Chou *et al*. [15]	42 years, M	Chest pain and productive cough	Pleural effusion and irregular thickness of the left pleura with small nodularities.	Pleural biopsy	Pleurectomy	Radiotherapy and chemotherapy for recurrent disease.	5	>14
	27 years, M	Dry cough, hoarseness, and chest tenderness	Pleural mass and left hemidiaphragm paralysis	Partial pleurectomy: fibrotic lesion measuring 3.7 × 2.0 × 2.0 cm in size in the left pleura of the lung apex.	Pleurectomy	Postoperative radiotherapy and subsequent pleurectomy for recurrent disease. Adjuvant doxorubicin and cisplatin.	NR	≈18
Bevelaqua *et al*. [16]	22 years, M	Fever	Right pleural effusion	Thoracocentesis: serosanguineous exudative effusion negative for malignancy.	Excission of the mass, one-third of the diaphragm, a portion of the right lower lobe, and associated parietal pleura.	NR	NR	NR
				Exploratory thoracotomy: lobulated hemorrhagic mass originating from the diaphragm.				
Apolinário *et al*. [17]	47 years, M	Chest pain and dyspnea	Left pleural effusion	Cytological examination: negative for malignancy.	Pulmonary decortication and parietal pleurectomy	Postoperative doxorubicin	NR	≈5
				Bronchofibroscopy: negative for malignancy.				
Takenaka *et al*. [18]	62 years, M	Right chest pain and dyspnea	Diffuse pleural thickening with uptake at FDG-PET and right pleural effusion	Thoracoscopy with pleural biopsy: severe adhesion in the thoracic cavity.	Extrapleural pneumonectomy	Pazopanib for recurrent disease	1	≈3.5

Given the rarity of the disease and the lack of large prospective trials, no established standard treatment for EHE is available. The management of advanced and symptomatic disease is often very challenging since chemotherapeutic agents approved for soft tissue sarcomas have limited activity in EHE [19].

Two translocations are the most common gene alterations of EHE: *WWTR1-CAMTA1* gene fusion in about 90% of cases, and *YAP1-TFE3* gene fusion in about 10% of cases, seemingly associated with different clinical course [8].

Here we report a patient who had successfully treated with macroscopically radical surgery for symptomatic primary pleural EHE.

## Case report

**Fig. 1:**
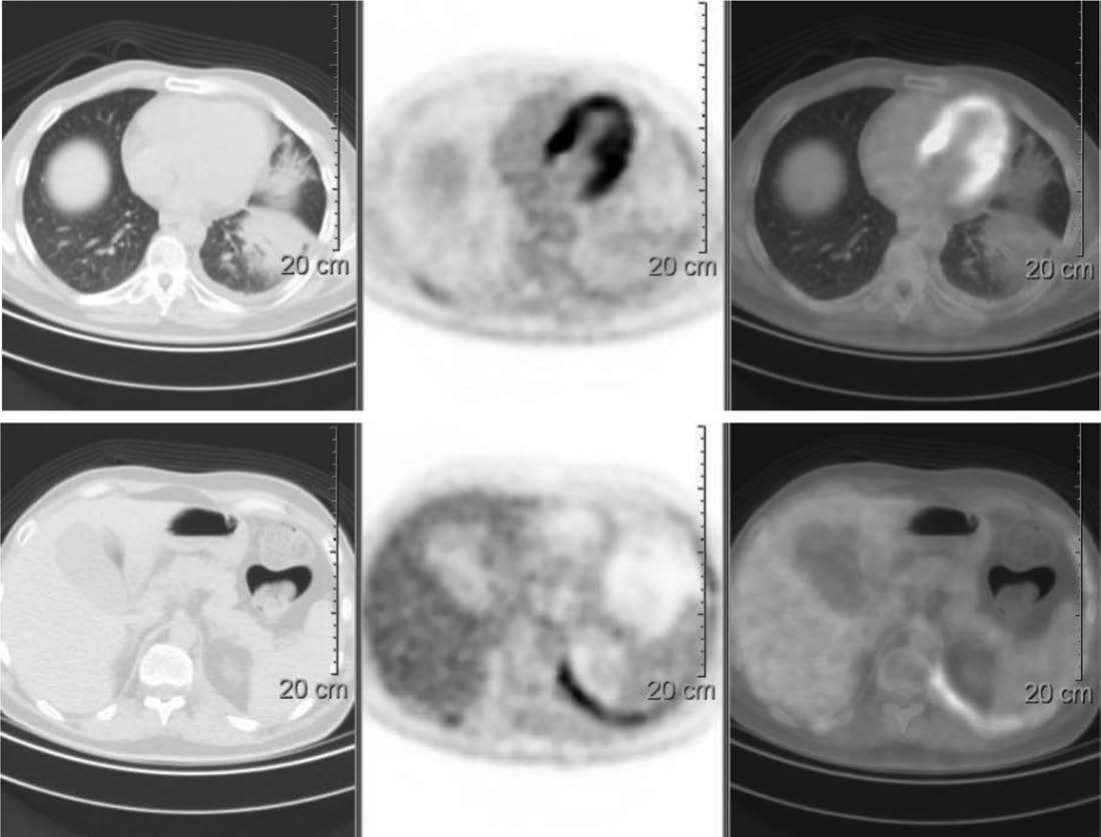
18F-FDG PET: preoperative mild diffuse tracer uptake in multiple areas of the left pleura.

**Fig. 2:**
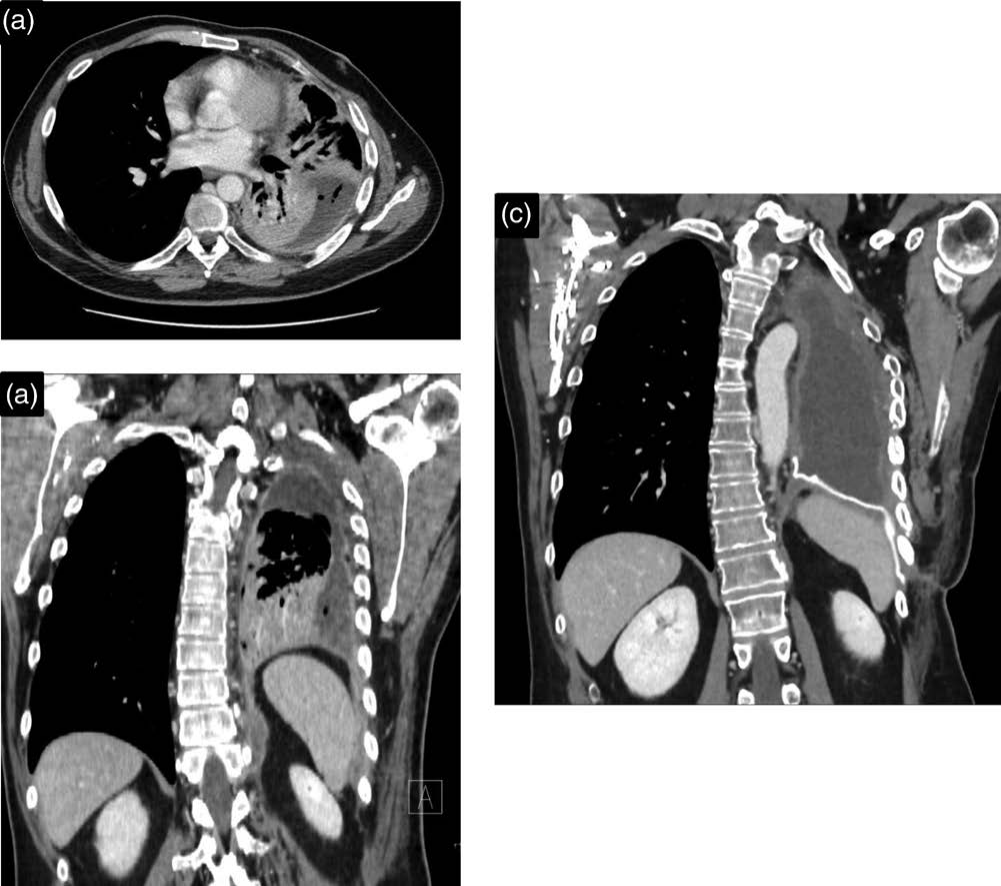
Preoperative contrast-enhanced CT scan images on axial and coronal planes (a) and postoperative contrast-enhanced CT scan image on coronal plane (b).

**Fig. 3:**
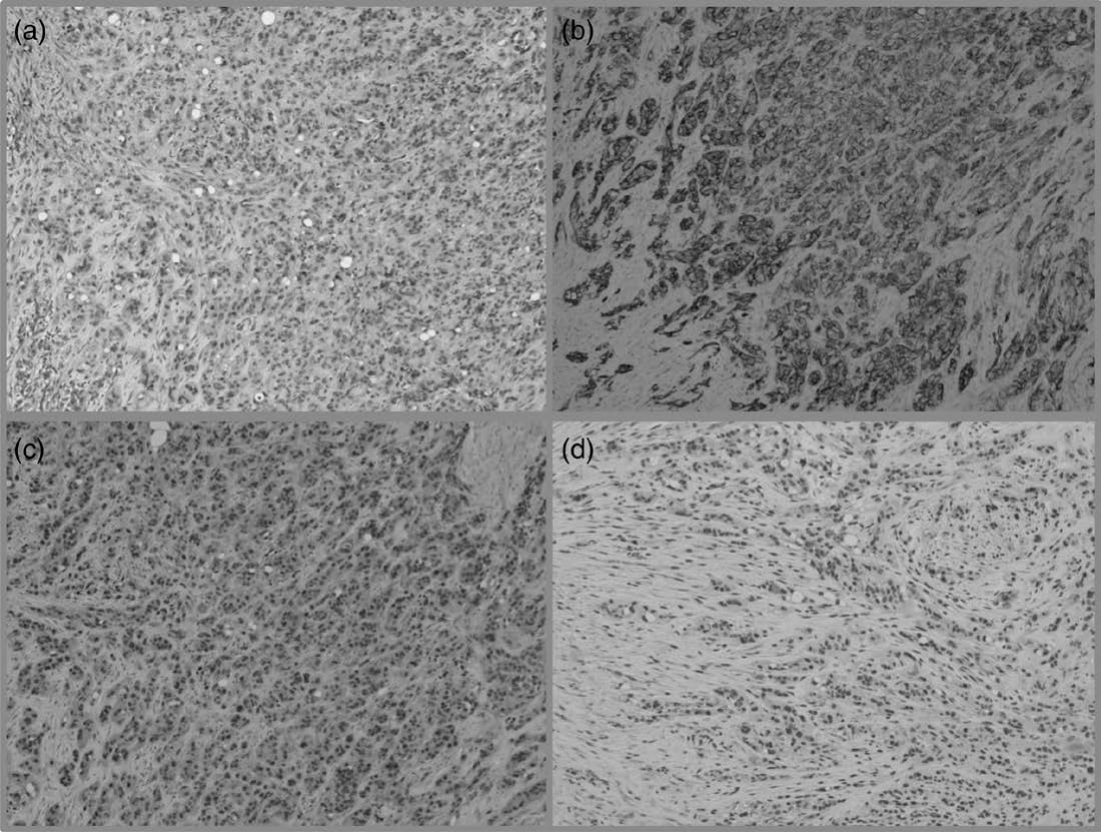
Histological examination. (a) Hematoxylin–eosin showing neoplastic proliferation consisting of nests and cords of epithelioid cells having glassy eosinophilic cytoplasm and oval vesicular nuclei (10×); widespread and intense positivity on immunohistochemistry for (b) CD31 (10×), (c) ERG (10×), (d) CAMTA1 (10×).

**Fig. 4:**
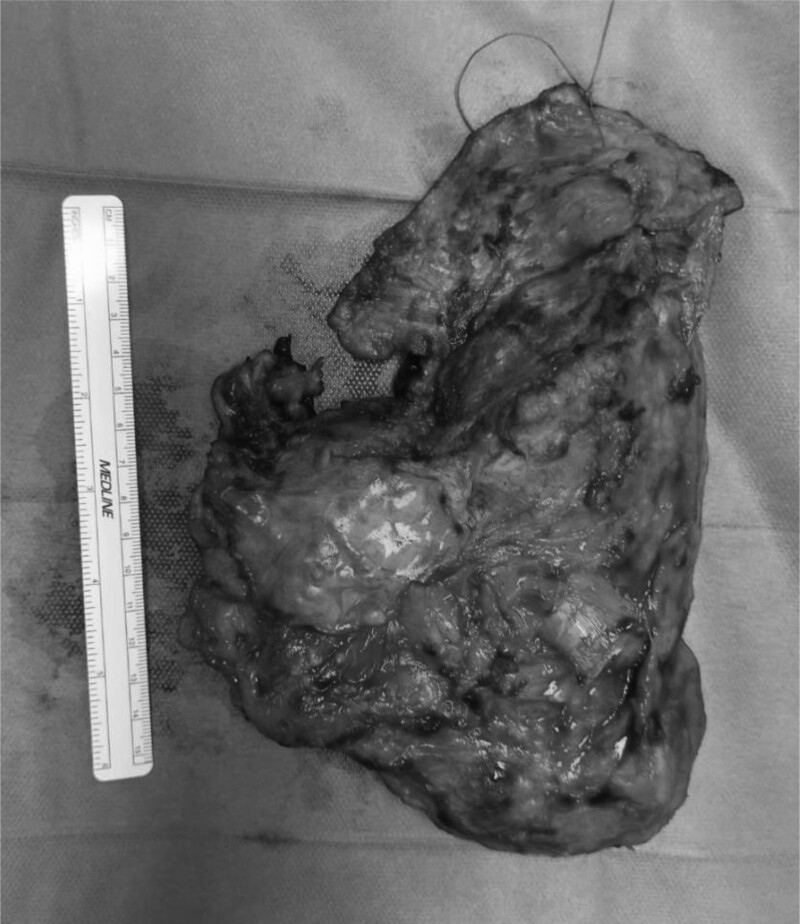
Surgical specimen: left lung with diaphragmatic flap. Neoplastic proliferation involves pleura, interlobar septa and diaphragm (total extension of 15 cm).

In April 2020, a 53-year-old Caucasian man came to our hospital, presenting chronic pain in the left hemithorax with transitory flare, particularly in the evening. A diagnosis of pleurisy was initially suspected and the patient received antibiotics, corticosteroids and no steroidal anti-inflammatory drugs with low improvement. The patient was then hospitalized due to a quick pain worsening. A computed tomography (CT) scan showed a very slight left pleural effusion, multiple bilateral ground glass pulmonary nodules with ‘tree-in-bud’ pattern and an inflammatory thickening in the left lower lobe. Thus, bronchoscopy with transbronchial needle aspiration was performed with negative results. In May 2020, an 18F-FDG PET showed a mild diffuse tracer uptake in the left pleura (Fig. 1). A few weeks later, the patient experienced a progressive worsening of pain requiring high doses of opioids. The clinical conditions were progressively getting worse and he started to develop systemic symptoms like severe fatigue, anemia, weight loss and uncontrolled pain. A new CT scan showed a left-sided pleural effusion with diffuse pleural thickening (Fig. 2). He underwent pleuroscopy, which revealed irregular thickness of the left pleura. Biopsies from parietal and diaphragmatic pleura showed an infiltrating neoplastic proliferation consisting of nests and cords of epithelioid cells having glassy eosinophilic cytoplasm and oval vesicular nuclei. Cytoplasmic vacuoles were evident. Immunohistochemistry showed diffuse positivity for CD31, ERG, CD34 and CAMTA1 and negative staining for cytokeratins AE1/AE3, STAT-6, TTF-1 and P40. The diagnosis of EHE was assessed. (Fig. 3) Due to the low response rate to chemotherapy in this disease, the worse prognosis determined by pleural involvement and the need for rapid symptom control, in July 2020, we discussed in a multidisciplinary fashion a surgical treatment in order to better symptoms control and to improve quality of life. The patient underwent extrapleural pneumonectomy in July 2020 (Fig. 4). On surgical specimen, histological examination showed pleural, lung, pericardial, diaphragmatic and peritoneal involvement. In the following months, the patient obtained excellent pain relief with progressive reduction of the opioid dose. He resumed active life and normal daily activities regaining the lost weight. Eight months after surgery, in March 2021, 18F-FDG PET and CT scans showed no radiological evidence of recurrent disease, although the patient had a slow worsening of painful symptoms.

## Surgical procedure

Surgery consisted in a left extrapleural pneumonectomy (Fig. 4). A first standard left posterolateral thoracotomy was performed along the course of the sixth rib. A subperiosteal resection of the sixth rib was then performed to provide wider exposure and to readily facilitate the initiation of the extrapleural dissection. Once the extrapleural plane was established the entire lateral dissection, both superiorly and inferiorly was done bluntly with a dissecting hand with subsequent placement of a chest retractor. Dissection then proceeded anteriorly both superiorly toward the apex of the chest and inferiorly toward the diaphragm using blunt and sharp techniques. Care was taken at the apex to avoid injury to the subclavian artery and vein and medially to preserve the internal mammary artery and vein dividing small adhesions, staying close to the pleural plane in the extrapleural fat. As dissection continued along the mediastinum, care was taken to avoid injury to the left vagus and left recurrent laryngeal nerve. The dissection continued posteriorly along the aorta, with particular attention to enter the correct plane in the preaortic region in order to avoid avulsing the intercostal vessels. Dissection was carried extrapleurally until the left main bronchus was clearly identified. Once the specimen has been circumferentially dissected from the chest wall, we performed a second lateral thoracotomy, entering the pleural space through the eight intercostal space and move the rib spreader to this interspace in order to provide better exposure to the diaphragm, after having continued the extrapleural dissection into the costophrenic sulcus. The diaphragm was incised at its anterior margin and extended in a circumferential fashion laterally and posteriorly with care being taken to avoid injury to the left vagus nerve and the esophagus at the hiatus and to preserve the underlying peritoneum which was swept away from the diaphragm with the aid of a sponge stick. The diaphragmatic muscle fibers were divided with cautery or simply avulsed from their insertion. The retractor was then moved back to the fifth intercostal space and the pericardium was entered antero-inferiorly and the pulmonary vessels were identified. The left main pulmonary artery was divided in its extrapericardial, extrapleural position using an endoscopic linear stapling device. The upper and lower left pulmonary veins were encircled within the pericardium and individually divided with a vascular stapling device. The pericardial resection was then completed posteriorly. Finally, the left main bronchus was dissected back to the carina and divided flush with the carina with a bronchial stapler, after having retracted the double lume endotracheal tube in trachea. So doing, the left bronchial stump retracted well back into the mediastinum and it was considered unnecessary for its buttressing. The surgical specimen was removed and a systematic lymph node dissection completed the resectional phase. The entire hemithorax was inspected for hemostasis. Reconstruction of the diaphragm was then carried out, moving the retractor to the lower intercostal incision. The diaphragm was reconstructed with a 2-mm thick PTFE patch (20 × 30 cm) secured to the chest wall laterally, anteriorly and posteriorly by the placement of interrupted monofilament sutures. Medially, the diaphragmatic path was sewn to the diaphragmatic edge of the divided pericardium and to the fibers of the crus. Then the pericardium was reconstructed with a 0.1-mm PTFE pericardial patch using a nonabsorbable monofilament suture. Fenestrations were created in the patch to prevent tamponade. Inferiorly, the patch was secured to the diaphragmatic patch with several interrupted monofilament sutures. The pleural space was irrigated with 2 liters of aqueous betadine solution. The chest was closed over a single basal drainage tube that had been connected to a balanced drain. The recovery was uneventful and the patient was discharged home in the 12th postoperative day.

## Discussion

Diagnosis and treatment of EHE remain a challenge. A watchful-waiting approach until disease progression could be a reasonable option mostly in patients with multifocal and/or multicentric presentation without symptoms and/or serosal effusions. Some authors indeed reported long-lasting stable disease (SD) with observation only or even a spontaneous regression of metastatic lesions [1,8,20]. Although no standard treatment has been established, complete surgical excision should be considered in localized disease. In a case series of patients (*n* = 32) treated at The Royal Marsden Hospital, 20% of whom underwent surgical resection, surgery and liver only disease were the two main factors associated with long-term survival outcomes [21]. Unfortunately, more than two-thirds of patients had a multifocal/multicentric disease, no longer suitable for local treatments. In these cases, medical therapy has rarely been associated with significant clinical benefits. Overall, the response rate was low with chemotherapy, but sometimes an improvement in symptoms was observed, including relief from pleuritic pain or irritable cough. To date paclitaxel, gemcitabine or anthracycline-based combinations, mostly used in angiosarcoma, are the most commonly used cytotoxic agents [19,21–24]. Anecdotal responses to other chemotherapeutic agents (i.e. carboplatin etoposide and cyclophosphamide) were reported, but evidence is limited and results are controversial [19,25,26].

Immunomodulatory and antiangiogenic drugs seem to be the most promising treatment options, but evidence derives primarily from retrospective case series and case reports. Interferon and thalidomide were initially used with conflicting results [19,24,27,28]. A remarkable benefit was observed with sirolimus in a series of patients with progressive EHE treated within the Italian Rare Cancer Network. Sirolimus was demonstrated to be feasible and effective in terms of disease control [6]. In a recent update of this series including 37 patients evaluable for response, disease control rate (DCR) was 86.5% with 4 patients who experienced a partial response (PR) (ORR 10.8%). Median progression-free survival (PFS) was 13 months and median overall survival (OS) was 18.8 months. This study confirmed pleural effusion as a predictor of poor prognosis, with median PFS of 4.8 months (*P* < 0.0001) and OS of 10.6 months (*P* < 0.0001) [29].

Given the vascular origin of EHE, the antiangiogenic strategy was considered a rational approach and the activity of several drugs has been studied in the past. The French Sarcoma Group conducted a phase II trial with sorafenib in 15 patients with EHE, of whom 10 in first-line treatment and 5 in subsequent lines. Only one patient in this group had a primary pleural EHE. Sorafenib showed a modest clinical activity with 9-month PFS rate of 30.7% and 2-year OS rate of 26%. Two PR were reported [30]. Other antiangiogenics including axitinib, semaxanib, pazopanib and sunitinib were used in a retrospective case series of Yousaf *et al*. Although no responses were observed in pretreated patients, disease stabilization and symptom control were reported [21]. Prolonged SD with the use of pazopanib was also described in two case reports [31,32], with PFS of 8 years and 2 years, respectively. In 2009, pazopanib received Food and Drug Administration and than European Medicines Agency approval for the treatment of pretreated advanced soft tissue sarcoma, including EHE [33]. A retrospective study collecting data from patients included in the European Organization for Research and Treatment of Cancer (EORTC) phase II and III trials or treated in clinical practice at EORTC centers was conducted. Among patients with EHE, pazopanib provided a DCR of 60% (6 out of 10 patients) with a median PFS of 26.3 months; [34] conversely the recently published observational retrospective case series study within the World Sarcoma Network showed no responses in 12 patients treated with pazopanib with a median PFS and a median OS of 2.9 and 8.5 months, respectively [24]. Bevacizumab has also shown clinical activity in the treatment of vascular sarcoma. In a multicenter phase II study including 7 patients with advanced EHE treated with bevacizumab, 2 patients achieved a PR and 4 an SD (ORR of 29% and DCR of 86%) [35].

Although our patient had several negative prognostic factors as pleuro-pulmonary disease, pleural effusion and systemic symptoms, he greatly benefited from palliative surgery. Nevertheless, the management of patients with pleural EHE remains challenging and extended palliative surgery, as in our case, cannot be routinely proposed. Only a few cases of patients treated with palliative surgery for pleural EHE have been reported (Table 1). Overall, these patients had different clinical courses and survival after surgery ranged from a few months to more than 2 years. Therefore, given the unpredictable clinical behavior of the disease, after a multidisciplinary discussion in sarcoma tumor board, palliative surgery could be an option in selected patients in centers with high volume and expertise in soft tissue sarcoma.

## Acknowledgements

### Conflicts of interest

There are no conflicts of interest.
